# Draft Genome Sequence of the Biosurfactant-Producing Bacterium *Gordonia amicalis* Strain CCMA-559, Isolated from Petroleum-Impacted Sediment

**DOI:** 10.1128/genomeA.00894-13

**Published:** 2013-10-31

**Authors:** Daniela F. Domingos, Bruna M. Dellagnezze, Paul Greenfield, Luciana R. Reyes, Itamar S. Melo, David J. Midgley, Valéria M. Oliveira

**Affiliations:** Microbial Resources Division, Research Center for Chemistry, Biology and Agriculture (CPQBA), Campinas University (UNICAMP), Campinas, São Paulo, Brazila; CSIRO Mathematics, Informatics and Statistics, North Ryde, NSW, Australiab; Laboratory of Environmental Microbiology, EMBRAPA Environment, Jaguariúna, São Paulo, Brazilc; CSIRO Animal Food and Health Sciences, North Ryde, NSW, Australiad

## Abstract

*Gordonia amicalis* strain CCMA-559 was isolated from an oil-contaminated mangrove swamp and shown to produce biosurfactants. This strain is a strict aerobe that readily degrades an array of carbon sources, including *N*-acetylglucosamine, cellobiose, Tween 80, and 4-hydroxybenzoic acid, and, like other *G. amicalis* strains, likely desulfurizes dibenzothiophene.

## GENOME ANNOUNCEMENT

*Gordonia amicalis* strain CCMA-559 was obtained from mangrove swamp sediment contaminated with crude oil and was isolated as described in Domingos et al. (1). The strain was shown to produce industrially important biosurfactants and bioemulsifiers and was thus subjected to genomic sequencing.

The shotgun sequencing of the *G. amicalis* CCMA-559 genome was performed using Illumina HiSeq 2000 technology. The resulting 100-bp paired-end sequences were corrected with Blue (http://www.bioinformatics.csiro.au/blue) and assembled by using Velvet 1.2.07 (*k* = 57) (2). The draft genome, as submitted to GenBank, was 5,179,574 bp in length and comprised 117 contigs (>200 bp in size), with a mean contig size of 44,269 bp, a median size of 5,879 bp, and maximum length of 380,987 bp. The mean GC content of the genome was 65.09%, and genome coverage depth was approximately 1,000×. Fifty-six short contigs (each <200 bp, totaling 8,819 bp) were excluded from the GenBank submission; however, these short sequences are available in the annotation at the Integrated Microbial Genomes Expert Review (IMG ER) website (https://img.jgi.doe.gov/er/). Annotation was performed using the IMG ER pipeline (3), which predicted a total of 4,736 protein-coding genes and 63 structural RNAs. Based on comparison of the 16S rRNA gene, *G. amicalis* CCMA-559 was 100% identical (over 1,400 bp) with *G. amicalis* IEGM, the type strain for the species, isolated from garden soil in Russia (4).

In terms of physiology, the IEGM and CCMA-559 strains of *G. amicalis* are broadly similar. Both have genes for the desulfurization of dibenzothiophene and use some similar carbon sources for growth. For example, both strains are capable of growth on starch and polyols, such as glycerol. There are, however, some notable differences between the strains. *G. amicalis* IEGM was not capable of growth on *N*-acetyl-d-glucosamine, cellobiose, and Tween 80 (4). In contrast, Biolog EcoPlate data for *G. amicalis* CCMA-559 indicate growth on *N*-acetylglucosamine, cellobiose, and Tween 80. To better understand the mechanisms for observed growth, genes from *G. amicalis* CCMA-559 were submitted to the dbCAN pipeline (5) for the classification of carbohydrate active genes. In combination with the annotation at IMG ER, data from dbCAN revealed the presence of at least one cellobiase (β-glucosidase) and numerous lipases/esterases in the genome of *G. amicalis* CCMA-559 (from carboxylesterase [CE] families 1, 3, 5, 6, 10, and 14), which likely facilitate growth on cellobiose and Tween 80, respectively. Growth on *N*-acetylglucosamine is presumably facilitated by the activity of acetylglucosaminidases in the genome of *G. amicalis* CCMA-559. We were unable to locate this gene in the annotation or dbCAN data; however, acetylglucosaminidases have been previously described for *Gordonia bronchialis*. In addition, growth on the monoaromatic compound 4-hydroxybenzoic acid was observed for *G. amicalis* CCMA-559 and is presumably facilitated by genes in the draft assembly that encode dioxygenases. Investigation of the genomes of this and other *Gordonia* species may yield further insights into their considerable metabolic potential.

### Nucleotide sequence accession numbers. 

This whole-genome shotgun project has been deposited at DDBJ/EMBL/GenBankunder the accession number AWTB00000000. The version described in this paper is version AWTB01000000.
